# Internet videoconferencing for patient–clinician consultations in long-term conditions: A review of reviews and applications in line with guidelines and recommendations

**DOI:** 10.1177/2055207619845831

**Published:** 2019-04-23

**Authors:** Agnieszka Ignatowicz, Helen Atherton, Celia Janine Bernstein, Carol Bryce, Rachel Court, Jackie Sturt, Frances Griffiths

**Affiliations:** 1 Institute of Applied Health Research, University of Birmingham, Birmingham, United Kingdom; 2 Warwick Medical School, The University of Warwick, Coventry, United Kingdom; 3 Florence Nightingale Faculty of Nursing, Midwifery and Palliative Care, King’s College London, London, United Kingdom; 4 Centre for Health Policy, School of Public Health, University of the Witwatersrand, Johannesburg, South Africa

**Keywords:** Internet videoconferencing, long-term conditions, review of reviews

## Abstract

**Background:**

The use of internet videoconferencing in healthcare settings is widespread, reflecting the normalisation of this mode of communication in society and current healthcare policy. As the use of internet videoconferencing is growing, increasing numbers of reviews of literature are published.

**Methods:**

The authors conducted a review of the existing reviews of literature relating to the use of internet videoconferencing for consultations between healthcare professionals and patients with long-term conditions in their own home. The review was followed with an assessment of United Kingdom National Institute for Health and Clinical Excellence guidelines for patient care in the context of common long-term illnesses to examine where videoconferencing could be implemented in line with these recommendations.

**Results:**

The review of reviews found no formal evidence in favour of or against the use of internet videoconferencing. Patients were satisfied with the use of videoconferencing but there was limited evidence that it led to a change in health outcomes. Evidence of healthcare professional satisfaction when using this mode of communication with patients was limited. The review of guidelines suggested a number of opportunities for adoption and expansion of internet videoconferencing. Implementing videoconferencing in line with current evidence for patient care could offer support and provide information on using a communication channel that suits individual patient needs and circumstances. The evidence base for videoconferencing is growing, but there is still a lack of data relating to cost, ethics and safety.

**Conclusions:**

While the current evidence base for internet videoconferencing is equivocal, it is likely to change as more research is undertaken and evidence published. With more videoconferencing services added in more contexts, research needs to explore how internet videoconferencing can be implemented in ways that it is valued by patients and clinicians, and how it can fit within organisational and technical infrastructure of the healthcare services.

## Introduction

The use of internet videoconferencing in healthcare settings is widespread, particularly to support contact with patients in remote and rural areas across the world.^1,2^ Previous studies on the use of videoconferencing have reported increased benefits in patient care in terms of reduced travel to hospital sites and convenience in consulting with clinicians from the patients’ own homes,^3,4^ particularly for those with long-term conditions.^5–7^ Increasingly, policymakers in the United Kingdom (UK) and elsewhere, are encouraging the use of internet videoconferencing with patients in routine healthcare settings,^8^ reflecting the normalisation of videoconferencing in society and current policy.^9,10^ In the UK, the government has been investing in the infrastructure for digital communication. The software has been rolled out to allow Skype to be used safely and securely in the specialist clinical settings.^11^ However, there has been some concern that Skype may pose regulatory and logistical challenges and may not be acceptable to patients and healthcare professionals. In our recent LYNC study (*Improving health outcomes for young people with long-term conditions: The role of digital communication in current and future patient-clinical communication*), we researched early adopters of other digital communication (such as email, text messages and mobile phones) from 20 National Health System (NHS) specialist clinical teams from across England and Wales and provided evidence on cost, patient safety, ethics and patient experience.^12,13^ The LYNC study found that some clinicians were using Skype with patients but were not prepared to openly admit it because of information governance policies. To make evidence-based decisions, providers and policy makers need to know about acceptability, feasibility and cost for patient and health system, and how internet videoconferencing is best deployed alongside other forms of digital communication. As the use of Skype and other forms of videoconferencing has grown in recent years, many reviews of research evidence have been published to reflect this rise. With the plethora of reviews available, it may be difficult to access the appropriate evidence. Furthermore, where and how videoconferencing could be used in consonance with current practice and guidelines for patient care has rarely been explored.

Clinical pathways are tools used by health professionals to determine the best way to manage specific medical conditions according to the best available evidence.^14^ The pathways map out, in chronological order, the key activities in a healthcare process for specific patient populations.^15^ In the UK, the National Institute for Health and Clinical Excellence (NICE) produces guidelines for health, public health and social care practitioners. Guidelines are published on the NICE website with the summarised evidence and resources to help practitioners implement them. These guidelines are also included in the NICE pathways, online tools that include up-to-date advice, quality standards and related information, from preventing and managing specific conditions to improving health and managing medicines in different healthcare settings. In recent years, NICE has developed many new care pathways, including those for long-term conditions. For example, the pathway for type 2 diabetes includes specific guidelines on monitoring patient’s blood pressure and glucose, as well as on identifying and managing complications and individualised care. As such, these tools can be implemented to improve care delivery for patients. However, videoconferencing pathways are not currently represented in these policy documents.

In this paper, we summarise the existing reviews of literature relating to the use of internet videoconferencing. We follow this review with an assessment of NICE pathways for common long-term conditions and identify where, from our interpretation of these guidelines, internet videoconferencing could be an appropriate option for healthcare delivery. Arguably, it may provide advantages to patients, their clinicians and/or healthcare systems. Finally, we interpret the results from the literature and NICE guidelines review in light of findings from the LYNC study, examining how videoconferencing fits with the use of other digital communication media.

## Aims

The aims of this paper are:

1. To summarise the existing reviews of literature relating to the use of internet videoconferencing between patients with long-term conditions and their treating clinicians from the patient’s own home (or mobile device).
2. To review the NICE guidelines for long-term conditions (LYNC study conditions: psychosis and schizophrenia, HIV, diabetes, liver fibrosis, eczema, psoriasis, cancer, asthma, cystic fibrosis, arthritis, kidney and sickle cell disease).
3. To identify where, in the patient pathway, the use of videoconferencing might be possible and of advantage to the patient, their clinician and/or the healthcare system.

## The review of reviews

### Methods

In collaboration with a trained information specialist (RC), a set of searches was developed that aimed to capture reviews on the use of videoconferencing for clinical communication. Firstly, using search terms ‘skype’ ‘videoconferenc*’ ‘video-conferenc*’ ‘Google AND (talk or hangouts)’ in any field, we searched the *EndNote* database of results from the review of systematic reviews undertaken in 2014 as part of the LYNC study.^13^ This database included records from sensitive searches of *MEDLINE*, *Embase*, *PsycINFO*, *Science Citation Index* and *Social Science Citation Index*, published from 2009 onwards. We updated this search in *MEDLINE* in June 2017 using more synonyms and brand names. We then undertook a further search combining thesaurus and free-text terms for the concepts of ‘internet videoconferencing technology’ and ‘reviews’ in order to capture non-systematic literature reviews. Finally, the results of an update scoping search for primary studies involving internet videoconferencing, undertaken for the LYNC study, were also checked for additional reviews. Details of the search strategies and sources used are provided in supplementary file 1.

Results from the searches in 2014 were screened independently by one reviewer (HA) who rated the eligibility of the records to confirm relevant papers. Abstracts and full-text papers were then reviewed by two authors (HA and CJB) to determine studies to be included for full review. From each included paper, using a standardised form, two reviewers extracted information about (1) the purpose of the study; (2) patients and participants; (3) the clinical application area; (4) the study design; (5) the country, or countries, where the study was conducted; and (6) whether the study findings supported the clinical use of videoconferencing and Skype. The process was then repeated for papers from the updated searches in 2017 by AI and CB.

This review included literature reviews, systematic reviews and meta-analyses. Studies were included if they: (1) included reviews conducted in healthcare settings where internet video use was with a patient in their own home or on a mobile device; and (2) focused on patients with long-term illnesses. We included English language articles only, and only those published since 2009. We excluded reviews of reviews and those review articles that focused on patients with multiple long-term illnesses.

The quality of each study was appraised by reviewers using the Assessment of Multiple Systematic Reviews (AMSTAR) checklists.^16^ Each checklist criterion was scored as 1 (checklist criterion satisfied); or 0 (checklist criterion not satisfied, or unclear) yielding a quality score across all criteria for each study in the range of 0 (worst) to 11 (best). Reporting was guided, where applicable, by PRISMA guidelines.^17^ The data are presented as a narrative synthesis of the findings from the identified reviews.

## Results

**Figure 1.**
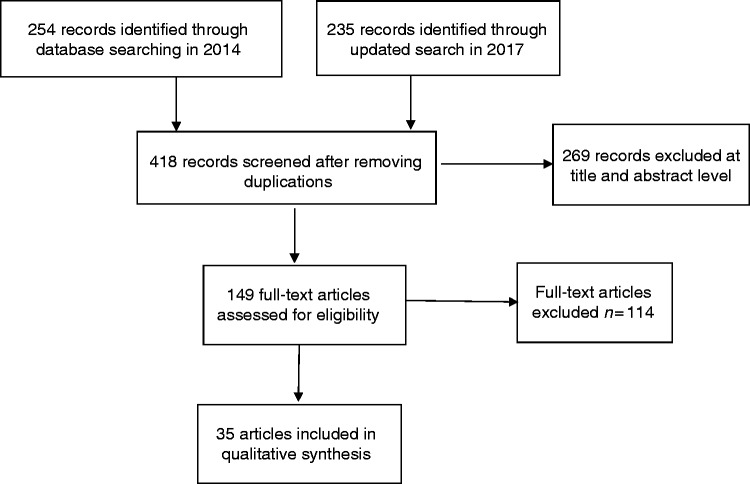
Study PRISMA flow diagram.

A total of 489 relevant studies were identified in the searches, which included several systematic reviews. After screening, a total of 149 abstracts were selected for full-text review and 35 review articles were included in the review (Figure 1).

## Review characteristics

The characteristics of the included reviews are summarised in Table 1. These covered a wide range of long-term conditions, including: heart failure, depression, schizophrenia, stroke, asthma, spinal cord injury, and chronic pain. Of 35 articles included in this review, 25 were reviews or systematic reviews. Overall, eight looked at internet videoconferencing exclusively,^7,18–24^ with the remainder examining a range of telehealth interventions including videoconferencing. Only one review of the clinical use of Skype was identified.^7^ Among the videoconferencing exclusive reviews, there were five that included more than 25 studies in their review.^7,18,21,25,26^ In 24 of the included reviews, forms of internet videoconferencing were compared with a face-to-face consultation or usual care.

**Table 1:** Characteristics of included reviews.

Author	Year	Review type	Number of papers/studies included	Participants and conditions	Intervention	Comparison
Armfield et al.^7^	2015	Systematic review	27	Patients with chronic conditions	Videoconferencing (Skype)	Usual care
Backhaus et al.^18^	2012	Systematic review	65	Patients with mental illness	Videoconferencing	Face-to-face consultation
Boisvert et al.^68^	2010	Systematic review	8	Patients with autism spectrum disorders	Telepractice (communication technologies such as laptops, videoconferencing and the internet)	Face-to-face consultation
Conway et al.^28^	2014	A sub-analysis of a previously published systematic review and meta-analysis^42,43^	25	Patients with heart failure	Four specific technologies (structured telephone calls, videophone, interactive voice response, telemonitoring)	Usual care
De Weger et al.^19^	2013	Literature review	18	Patients with mental illness	Videoconferencing	Face-to-face consultation
Dorstyn et al.^69^	2013b	Systematic review	7	Patients with spinal cord injury	Telecounselling (telephone and internet, including videoconferencing)	Information-only, usual care
Duncan et al.^70^	2014	Literature review	19	Young patients with mental illness	Videoconferencing	Face-to-face consultation
Garcia-Lizana and Munoz- Mayorga^31^	2010a	Systematic review	10	Patients with mental illnesses	Telepsychiatry (videoconferencing)	Face-to-face consultation
Garcia-Lizana and Munoz-Mayorga^32^	2010b	Systematic review	10	Patients with depression	Information communication technologies (e.g. website, internet programs, email, videoconferencing, and computer-telephone integrated system)	Face-to-face consultation
Gloff et al.^25^	2015	Review	29	Children and adolescents with mental illness	Telehealth (videoconferencing)	Face-to-face consultation
Hilty et al.^26^	2013	Review of literature	39	Patients with mental illness	Telehealth (videoconferencing)	Face-to-face consultation
Kasckow et al.^45^	2014	Systematic review	18	Patients with schizophrenia	Telepsychiatry (telephone, video or internet-based)	Reduced telephone call exposure, usual care, treatment as usual, face-to-face multifamily groups
Kitamura et al.^36^	2010	Systematic review	19	Patients with cancer	Video consultation	Face-to-face consultation
Mars et al.^39^	2012	A review of the literature	13	Psychologists or psychiatrists dealing with prisoners with mental illnesses	Forensic telepsychiatry (videoconferencing)	Face-to-face consultation
Martin et al.^40^	2011	Systematic review	12	Young people with mental illnesses	Networked Communication Interventions (email/web-based diary, video- or teleconferencing, and virtual reality)	Waiting list controls, face-to-face consultation
McGeary et al.^71^	2013	Meta-analysis	10	Patients with chronic pain	Telehealth (interactive and self-help websites, internet, telephone, internet and telephone, video- or teleconferencing, wireless biofeedback)	Face-to-face consultation, treatment as usual, waiting list controls
McLean et al.^29^	2010	Systematic review	21	Patients with asthma	Telehealth (telephone, videoconferencing, internet, other networked technologies, SMS, combination of SMS and the internet)	Face-to-face consultation, educational approaches (e.g. leaflets), usual care plans
McLean et al.^46^	2010	Review	Not stated	Patients with chronic conditions	Telehealthcare (telephone, videoconferencing, internet)	Face-to-face consultation
Nelson et al.^20^	2011	Review of literature	Not stated	Children and adolescents with mental illnesses	Videoconferencing	Usual care
Neubeck et al.^48^	2009	Systematic review	11	Patients with coronary heart disease	Telehealth interventions (telephone, internet)	Usual care
Paing et al.^72^	2009	Review	Not stated	Children and adolescents with mental health illnesses	Telemedicine	Face-to-face consultation
Peeters et al.^47^	2011	Systematic review	9	Patients at home and patients with chronic conditions	Video communication	Usual care at home
Peterson^73^	2014	Systematic review	14	Patients with Type I diabetes mellitus	Mobile tools (internet, mobile, mobile and internet, phone, videoconferencing and phone)	Unspecified
Ramos-Rios et al.^74^	2012	Review of literature	Not stated	Elderly patients with psychiatric illnesses	Telepsychiatry (videoconferencing)	Face-to-face consultation
Schleg et al.^41^	2015	Systematic review	40 studies/45 articles	Patients with anorexia and bulimia nervosa and their carers	Technology-based interventions (computer, videoconferencing, vodcasts, email, mobile/SMS, internet)	Waiting list controls, without intervention, face-to-face consultation, video or brochure controls, Beating Eating Disorders intervention
Sharp et al.^21^	2011	A review of the literature	33	Patients with psychosis	Videoconferencing	Face-to-face consultation
Shore^34^	2013	Review	Not stated	Patients with psychiatric illnesses	Telepsychiatry (videoconferencing)	Face-to-face consultation
Simpson and Reid^22^	2014	Systematic review	23	Patients with mental illnesses	Videoconferencing	Face-to-face consultation
Siriwardena et al.^35^	2012	A review of the literature	27	Patients with type I or II diabetes mellitus	Telemedicine (videoconferencing, mobile phone, telephone, feedback letters with or without telemonitoring)	Usual care, in-person health education, diabetes education group via videoconferencing with no follow up, telemonitoring using web application to upload blood glucose levels, controls received little feedback about blood glucose levels, waiting list controls, no intervention, blood glucose levels communicated over the telephone, telemonitoring only
Slone et al.^75^	2012	Review of literature	35	Children and adolescents with mental health	Telepsychology (videoconferencing, Internet, telephone)	Face-to-face consultation
Sucala et al.^33^	2012	Systematic review	11	Patients with mental illnesses	All text-based asynchronous and/or synchronous communicative e-therapies (email, website postings, website exchanges, email and chat, website postings and email, chat)	Face-to-face consultation
Van den Berg et al.^44^	2012	Systematic review	68	Older patients	Telemedicine (telemedical devices to measure vital signs, telephone, short messages, videoconferencing and combinations of these, interactive systems only in combinations with the aforementioned modalities)	Usual care, face-to-face consultation, self-management, health education, manual approaches, additional controls relating to the specific included studies
Van Allen et al.^23^	2011	Review	9	Children and adolescents with chronic illnesses	Videoconferencing/teleconferencing	Face-to-face consultation
Wile and Pringsheim^24^	2013	Systematic review and meta-analysis	8	Patients with Tourette Syndrome	Telehealth (videoconferencing)	Face-to-face consultation
Zhai et al.^27^	2014	Systematic review and meta-analysis	47 papers/35 studies	Patients with Type II diabetes mellitus	Telemedicine (websites, internet, videoconferencing, telephone-based, internet transmissions)	Usual care, face-to-face, diabetes self-management program

Overall, the methodological quality of included reviews was poor (Table 2). Only six reviews were methodologically strong,^7,22,26–29^ with the remaining 31 obtaining a score of 6 or below, thus being of poor quality. The most common methodological weaknesses were limited details of the included study characteristics, such as clinical outcomes, participants’ demographics and potential biases in the selection of articles. Within the included reviews, low and variable uptake and the cost of establishing videoconferencing services across primary studies were often identified as a limitation.

**Table 2.** Quality assessment of included reviews.

Author	Year	AMSTAR score (n/11)	Comments
Armfield et al.^7^	2015	11	Study matched all the criteria on AMSTAR. There were no methodological limitations to report.
Backhaus et al.^18^	2012	3	The authors refer to a protocol but do not provide a link to one, it is unclear whether the authors independently screened abstracts/titles and extracted data, a diagrammatic search strategy and publication dates not provided, grey literature not included, there is some detail missing concerning the reference list of excluded studies, scientific quality not assessed or reported appropriately in the conclusion, publication bias not assessed.
Boisvert et al.^68^	2010	2	Protocol not provided, it is unclear whether the authors independently performed searches on supplementary material, a diagrammatic search strategy not provided, grey literature not included, there is some detail missing concerning the reference list of excluded studies, scientific quality not assessed or reported appropriately in the conclusion, the authors have not adequately explained why they could not combine results, publication bias not assessed.
Conway et al.^*28^	2014	10	The authors provide a non-accessible reference to their protocol.
De Weger et al.^19^	2013	3	Protocol not provided, it is unclear whether the authors independently screened abstracts/titles, grey literature not included, excluded studies not referenced, publication bias not assessed, conflict of interest not stated.
Dorstyn et al.^30^	2013a	6	Protocol not provided, it is unclear whether the authors independently screened titles and abstracts, excluded studies not referenced, publication bias not assessed, conflict of interest not stated.
Dorstyn et al.^69^	2013b	5	Protocol not provided, it is unclear whether the authors independently screened abstracts/titles and only one author extracted data, the authors refer to an appendix supposedly containing details of key search terms but do not provide a link, grey literature not included, excluded studies not referenced, publication bias not assessed.
Duncan et al.^70^	2014	2	Protocol not provided, it is unclear whether the authors independently performed searches, a diagrammatic search strategy not provided, grey literature not included, excluded studies not referenced, scientific quality not assessed or reported appropriately in the conclusion, publication bias not assessed.
Garcia-Lizana and Munoz-Mayorga^31^	2010a (online)	6	Protocol not provided, independent duplicate assessments conducted on data extraction only, grey literature not included, excluded studies not referenced, publication bias not assessed.
Garcia-Lizana and Munoz-Mayorga^32^	2010b	6	Protocol not provided, independent duplicate assessments conducted on data extraction only, grey literature not included, excluded studies not referenced, publication bias not assessed.
Gloff et al.^25^	2015	4	Protocol not provided, it is unclear whether the authors independently extracted data, it is unclear whether the authors searched for supplementary material, grey literature not included, excluded studies not referenced, scientific quality not assessed or reported appropriately in the conclusion, publication bias not assessed.
Hilty et al.^26^	2013	8	Protocol not provided, grey literature not included, publication bias not assessed.
Kasckow et al.^45^	2014	2	Protocol not provided, it is unclear whether the authors independently extracted data, it is unclear whether the authors searched for supplementary material and they do not provide a diagrammatic search strategy, grey literature not included, excluded studies not referenced, scientific quality not assessed or reported appropriately in the conclusion, the authors have not adequately explained why they could not combine results, publication bias not assessed.
Kitamura et al.^36^	2010	2	Protocol not provided, only one author screened titles/abstracts, diagrammatic search strategy not provided and supplementary material not searched, it is unclear whether the authors included grey literature, excluded studies not referenced, scientific quality not assessed or reported appropriately in the conclusion, publication bias not assessed, conflict of interest not stated.
Mars et al.^39^	2012	1	Protocol not provided, it is unclear who screened titles/abstracts and extracted data and no information is provided as to whether these activities were conducted independently or whether any disputes were resolved by a third reviewer, it is unclear whether the authors searched for supplementary material, publication dates and a diagrammatic search strategy not provided, grey literature not included, excluded studies not referenced, scientific quality not assessed or reported appropriately in the conclusion, the authors have not adequately explained why they could not combine results, publication bias not assessed, conflict of interest not stated.
Martin et al.^40^	2011	6	Protocol not provided, it is unclear whether the authors independently screened titles/abstracts and extracted data, grey literature not included, there is some detail missing concerning the reference list of excluded studies, publication bias not assessed.
McGeary et al.^71^	2013	6	The authors refer to an unpublished protocol but do not provide a link to one, it is unclear whether the authors independently screened titles/abstracts and independently extracted data, excluded studies not referenced, the authors mention using Egger’s regression to assess publication bias but do not provide any statistical data, conflict of interest not stated.
McLean et al.^29^	2010	11	Study matched all the criteria on AMSTAR. There were no methodological limitations to report.
McLean et al.^46^	2011	3	Protocol not provided, it is unclear who screened titles/abstracts and extracted data and no information is provided as to whether these activities were conducted independently or whether any disputes were resolved by a third reviewer, it is unclear whether the authors searched for supplementary material, publication dates and a diagrammatic search strategy not provided, excluded studies not referenced, scientific quality not assessed or reported appropriately in the conclusions, publication bias not assessed.
Nelson et al.^20^	2011	2	Protocol not provided, search terms not included, it is unclear whether the authors independently extracted data, grey literature not included, excluded studies not referenced, the authors have not adequately explained why they could not combine results, scientific quality not assessed or reported appropriately in the conclusions publication bias not assessed.
Neubeck et al.^48^	2009	4	Protocol not provided, search terms not provided, there is some detail missing concerning the reference list of excluded studies, the authors mention that they assessed methodological quality using the Jadad score in order to exclude studies with a rating of less than 2 but do not provide scores for each of the included studies, scientific quality reported inappropriately in the conclusion, publication bias not assessed, conflict of interest statement does not include details of any possible funding source(s).
Paing et al.^72^	2009	5	Protocol not provided, search terms not included, it is unclear whether the authors independently extracted data, grey literature not included, excluded studies not referenced, the authors have not adequately explained why they could not combine results, publication bias not assessed.
Peeters et al.^47^	2011	5	Protocol not provided, it is unclear whether the authors independently extracted data, grey literature not included, excluded studies not referenced, the authors have not adequately explained why they could not combine results, publication bias not assessed.
Peterson^73^	2014	3	Protocol not provided, study selection and data extraction performed by one person only, supplementary material and grey literature not included, excluded studies not referenced, scientific quality not assessed for each included study or reported appropriately in the conclusion, publication bias not assessed.
Ramos-Rios et al.^74^	2012	7	Protocol not provided, it is unclear who screened titles/abstracts and extracted data, publication dates and a diagrammatic search strategy not provided, excluded studies not referenced, scientific quality not assessed or reported appropriately in the conclusions, publication bias not assessed.
Schlegl et al.^41^	2015	5	Protocol not provided, it is unclear whether the authors independently screened titles/abstracts, independently extracted data, and resolved disputes with a third reviewer, grey literature not included, excluded studies not referenced, publication bias not assessed, conflict of interest statement does not include details of any possible funding source(s).
Sharp et al.^21^	2011	2	Protocol not provided, it is unclear whether the authors independently screened titles/abstracts, independently extracted data, and resolved disputes with a third reviewer, a diagrammatic search strategy not provided, grey literature not included, excluded studies not referenced, scientific quality not assessed for each included study or reported appropriately in the conclusion, publication bias not assessed, conflict of interest statement does not include details of any possible funding source(s).
Shore^34^	2013	1	Protocol not provided, no information is provided on study selection, data extraction, search strategy, the inclusion of grey literature or excluded studies, scientific quality not assessed for each included study or reported appropriately in the conclusion, no information is provided on how the author combined the results, publication bias not assessed, conflict of interest statement does not include details of any possible funding source(s).
Simpson and Reid^22^	2014	8	Protocol not provided, it is unclear whether the authors independently screened titles/abstracts, independently extracted data, and resolved disputes with a third reviewer, publication bias not assessed.
Siriwardena et al.^35^	2012	2	Protocol not provided, it is unclear whether the authors independently screened titles/abstracts, independently extracted data, and resolved disputes with a third reviewer, only one database search was performed and no supplementary material was searched, grey literature not included, excluded studies not referenced, scientific quality not assessed for each included study or reported appropriately in the conclusion, no information is provided on how the authors combined the results, publication bias not assessed.
Slone et al.^75^	2012	3	Protocol not provided, it is unclear whether the authors independently screened abstracts/titles, extracted data and searched for supplementary material, grey literature not included, excluded studies not referenced, scientific quality not assessed for each included study or reported appropriately in the conclusion, publication bias not assessed.
Sucala et al.^33^	2012	5	Protocol not provided, it is unclear whether the authors initially independently screened titles/abstracts (they report only doing this for 56 potentially eligible studies) and included supplementary material in their literature searches, grey literature not included, excluded studies not referenced, publication bias not assessed.
Van den Berg et al.^44^	2012	1	Protocol not provided, it is unclear whether the authors independently screened abstracts/titles, extracted data and searched for supplementary material, grey literature not included, excluded studies not referenced, scientific quality not assessed for each included study or reported appropriately in the conclusion, the authors do not provide enough detail for why a narrative synthesis was used to pool findings, publication bias not assessed, conflict of interest statement does not include details of any possible funding source(s).
Van Allen et al.^23^	2011	4	Protocol not provided, search terms not included, it is unclear whether the authors independently screened abstracts/titles, extracted data and searched for supplementary material, grey literature not included, excluded studies not referenced, scientific quality not assessed for each included study or reported appropriately in the conclusion, publication bias not assessed.
Wile and Pringsheim^24^	2013	3	Protocol not provided, it is unclear whether the authors independently screened titles/abstracts, grey literature not included, excluded studies not referenced, the authors mention that they assessed methodological quality using the United States Preventive Services Task Force Quality Rating Criteria for Randomised Trials in order to exclude low quality studies but do not provide scores for each of the included studies, scientific quality reported inappropriately in the conclusion, publication bias not assessed, conflict of interest statement does not include details of any possible funding source(s).
Zhai et al.^27^	2014	9	Protocol not provided, grey literature not included.

*Some scores are based on the article’s original systematic review and meta-analysis.

## Patient, professional and health service delivery outcomes

The results from the included studies are presented in Table 3. The reviews commonly focused on effectiveness and outcomes for specific patient groups and conditions, and on patient satisfaction with this mode of communication. A total of six reviews found evidence of patient satisfaction and equivalence with face-to-face encounters and eight found improvement in at least one health outcome.

**Table 3.** Results of included reviews.

Author	Year	Results
Armfield et al.^7^	2015	**Patient outcomes:** Skype allows good communication between individuals and health professionals. Skype was more economical than face-to-face appointments with savings accruing from avoided travel.**Health professional outcomes:** Skype provides adequate quality to facilitate a diagnosis.**Health service delivery outcomes:** Skype is adequate for patients across the age spectrum, though more often for adult rather than for paediatric applications.
Backhaus et al.^18^	2012	**Patient outcomes:** Patients are satisfied with using videoconferencing in order to discuss their mental health conditions with a professional.**Health service delivery outcomes:** This modality is promising and feasible for those experiencing emotional disorders.
Boisvert et al.^68^	2010	**Health service delivery outcomes:** This modality is promising and feasible for patients with autism spectrum conditions.
Conway et al.^28^	2014	**Patient outcomes:** Structured telephone follow up and telemonitoring reduced heart failure-related hospitalisation admittances, but there was no conclusive evidence that this occurred with videophone.
De Weger et al.^19^	2012	**Health service delivery outcomes**: There is evidence to suggest that improvements in depressive symptoms, medication adherence, and remission rates do not differ greatly between videoconferencing and face-to-face groups. Videoconferencing may be more effective for anxiety-related disorders than for depression.
Dorstyn et al.^30^	2013a	**Patient outcomes:** Significant short-term improvements were associated with internet-based modalities. The evidence also indicates that in comparison with ‘information- or monitoring-only control conditions’, telecounselling is effective on its own. Limited data demonstrated longer-term improvements. However, this modality’s absolute effectiveness with in-person care is unknown.**Health service delivery outcomes:** Telecounselling is flexible, time-effective, and appeals to a variety of ages. It also diversifies the treatments patients receive.
Dorstyn et al.^69^	2013b	**Patient outcomes:** Telecounselling is promising for improving patients’ physical (e.g. pain) and emotional (e.g. depression) health in the short-term. The longer-term impact of this modality is unknown.**Health service delivery outcomes:** Telecounselling is time-efficient, practical, and appealing to patients.
Duncan et al.^70^	2014	**Health service delivery outcomes**: Telepsychological assessment yields similar results as face-to-face encounters for adult clients. Using videoconferencing to deliver psychotherapy appears favourable for rural youth who lack access to resources.
Garcia-Lizana and Munoz-Mayorga^31^	2010a (online)	**Patient outcomes:** Patients are satisfied with using videoconferencing technology. The limited data also suggests it is effective for improving patients’ symptoms and adherence to treatment. Telepsychiatry is also safe to use.**Health service delivery outcomes:** There is evidence to suggest that this modality improves service accessibility, provides educational services to patients, and saves time and money.
Garcia-Lizana and Munoz-Mayorga^32^	2010b	**Patient outcomes:** Videoconsulting increased patient satisfaction. The limited evidence suggests that this modality could improve symptoms when face-to-face care is unavailable.**Health service delivery outcomes:** Studies demonstrate that outcomes for videoconsulting are comparable with outcomes for the same therapy delivered in person.
Gloff et al.^25^	2015	**Patient outcomes:** Telemental health to reduce disparities and to improve the quality of child and adolescent mental healthcare.
Hilty et al.^26^	2013	**Patient outcomes:** Face-to-face services may be better for children and adolescents because of the novelty of the interaction, the impact of technology on the young person’s behaviour, the psychological and physical distance, and the authenticity of the family interaction.**Health service delivery outcomes:** Some studies reported reduced length of hospitalisation, better medication adherence, symptom reduction of disorders. Videoconferencing appears to be as effective as in-person care for feasibility, outcomes, age, and satisfaction with a single assessment and consultation or follow-up use.
Kasckow et al.^45^	2014	**Patient outcomes:** Telepsychiatry is promising and has shown to improve clinical outcomes in areas such as treatment adherence, symptoms, insight, perceived stress, and social support.**Health service delivery outcomes:** This modality is feasible, improves patient-staff communication, and decreases hospitalisation rates and visits to the emergency room. Limited data suggests that telepsychiatry is also cost-effective.
Kitamura et al.^36^	2010	**Patient outcomes**: Data suggests that videoconferencing is feasible and effective for assessing, monitoring, and managing patients with cancer. Patient satisfaction was reported. However, the methodological quality of the supporting evidence was generally weak and limited by unmatched controls, small samples, and inappropriate randomisation, making it difficult to ascertain the effectiveness of videoconferencing in this population.**Health service delivery outcomes:** Evidence points towards reductions in healthcare expenditure and travel/waiting times.
Mars et al.^39^	2012	**Patient outcomes:** While patient satisfaction for adjudicative competence has not been reported, there is evidence that prisoners are satisfied ‘with the use of videoconferencing for completing assessing tools’ (p. 245).**Health professional outcomes:** Clinician satisfaction for adjudicative competence has not been reported. Health professionals are less satisfied with videoconferencing.**Health service delivery outcomes:** Telepsychiatry is cost-effective, ‘improve(s) access to scarce specialist skills and reduce(s) transport of prisoners’ (p. 244). This modality also reduces the risk of harm to clinicians by enabling them to assess prisoners without entering a prison.
Martin et al.^40^	2011	**Patient outcomes:** Patients expressed satisfaction with using video conferencing. The data dealing with these modalities appears more rigorous and reliable. While networked technologies ‘offer patients a limited improvement in quality of life, continuity of care and access […] these gains were matched with concerns over privacy’ (p. e112).**Health professional outcomes:** Health professionals felt satisfied with using email and web-based technologies.**Health service delivery outcomes:** Limited data on financial implications makes it difficult to ascertain the cost-effectiveness of networked technologies.
McGeary et al.^71^	2013	**Patient outcomes:** Telehealth appears to produce beneficial results for patients undergoing pain treatment, but exact benefits are unknown. The evidence also indicates that this modality reduces pain intensity.**Health service delivery outcomes:** Limited data suggests that telehealth is cost-effective.
McLean et al.^29^	2010	**Patient outcomes:** Telehealth does not appear to produce a desired impact on quality of life for those with mild asthma. There is evidence of symptom improvement in telehealth trial arms where symptoms are managed more rapidly than the control arms.**Health service delivery outcomes:** Telehealth improves access to healthcare services and may also reduce costs and hospital admission rates, particularly for those with more severe asthma who are managed in secondary healthcare facilities.
McLean et al.^46^	2011	**Health service delivery outcomes:** Web-based clinical consultations, such as those for asthma or chronic obstructive pulmonary disease, or diabetes annual reviews, can replace routine visits such as face-to-face annual reviews. Overall, the evidence for cost-effectiveness is limited.
Nelson et al.^20^	2011	**Patient outcomes:** Videoconferencing is promising concerning patient satisfaction.**Health professional outcomes:** Studies examining therapeutic alliance have not found significant differences between therapeutic alliance developed in face-to-face and videoconferencing groups. Telemental health assessments are reliable, feasible and acceptable.**Health service delivery outcomes:** Limited data suggests that videoconferencing is cost-effective.
Neubeck et al.^48^	2009	**Patient outcomes:** Telehealth produces beneficial effects on reducing risk factors associated with coronary heart disease.**Health service delivery outcomes:** The scarce information provided by the reported trials on cost-effectiveness and delivery costs meant that the authors could not draw any conclusive statements.
Paing et al.^72^	2009	**Health service delivery outcomes:** The limited data indicates that telepsychiatry has the potential to be a useful treatment alternative for patients.
Peeters et al.^47^	2011	**Health service delivery outcomes:** The authors found no evidence to suggest that administering video communication to patients at home is cost-effective.
Peterson^73^	2014	**Patient outcomes:** The limited data indicates that mobile tools including video conferencing are a promising modality in the management of patients’ glycaemic levels.
Ramos-Rios et al.^74^	2012	**Health service delivery outcomes:** The use of telepsychiatry, and specifically, videoconferencing in psychogeriatric entails a number of challenges and a greater complexity than in the case of its application with other patients.
Schlegl et al.^41^	2015	**Patient outcomes:** Technology-Based Interventions including video conferencing may be beneficial for improving symptoms (e.g. purging) as well as treating and preventing eating disorders. This modality may also support carers looking after those with eating problems. No serious adverse effects were reported with using this modality.**Health professional outcomes**: The limited data indicates that there are differences between patients and therapists ‘in terms of adherence to therapeutic tasks, adherence to therapeutic goals, and therapeutic bond’ (p. 9).**Health service delivery outcomes:** Limited evidence suggests that the costs associated with telemedicine (including video) were lower, albeit still considerable. Cost-effectiveness was comparable to usual care.
Sharp et al.^21^	2011	**Patient outcomes:** Videoconferencing is relatively easy for patients with psychosis to use without exacerbating their symptoms. In fact, there is some evidence to suggest that the distance between patients and health professionals could reduce anxiety and over-simulation.**Health service delivery outcomes:** The data indicates a reduction in travel time for patients and health professionals, decreased hospitalisation rates, and an improvement in reaching those living in rural communities. It appears that videoconferencing produces more efficient healthcare.
Shore^34^	2013	**Health professional outcomes:** Videoconferencing is feasible and has gained popularity within psychiatry. It is important that psychiatrists learn how to effectively implement this technology and develop an understanding of the clinical, regulatory, and administrative issues associated with it. Psychiatrists should also generate an emergency protocol prior to caring for patients via videoconferencing and, if necessary, dialogue with them about their use or ownership of weapons and/or (il)legal substances. The psychiatrist should also reflect on their own communicative styles in order to ensure that they interact naturally with the patient as they would during face-to-face consultations.
Simpson and Reid^22^	2014	**Patient outcomes**: Patients rated the therapeutic alliance at least as high in the videoconferencing as in-person therapy.**Health professional outcomes:** Therapeutic alliance is high across diagnostic groups and interventions, and therapist-rated alliance is moderate to high in psychotherapy via videoconferencing.
Siriwardena et al.^35^	2012	**Patient outcomes:** Telemedicine (including video) is promising in the management of diabetes. Patients with non-insulin type II diabetes reported better clinical outcomes than insulin type I and type II patients. While two studies revealed negative metabolic improvement, one demonstrated that patients still found it helpful to contact their health professional over the telephone. Overall, patient satisfaction was high with telemedicine.**Health service delivery outcomes:** The data suggests that financial benefits are equal to usual care. This modality appears to reduce travel and in-clinic waiting times.
Slone et al.^75^	2012	**Health service delivery outcomes:** The evidentiary support for telepsychology for children and adolescents is encouraging but preliminary.
Sucala et al.^33^	2012	**Patient outcomes:** Three of the review’s included studies found that therapeutic alliance positively affected treatment outcomes and in some cases, reduced anxiety-related symptoms.Some studies also suggest that telemedicine interventions may provide similar clinical outcomes to those expected from in-person service delivery**Health service delivery outcomes:** E-therapy (which includes video) provides promising results for the delivery of mental health services. This modality also appears equivalent to face-to-face care in terms of therapeutic alliance, albeit the limited data precludes any firm conclusions.
Van Allen et al.^23^	2011	**Patient outcomes:** Young patients express satisfaction with using videoconferencing.**Health professional outcomes:** Some studies also suggest that telemedicine interventions may provide similar clinical outcomes to those expected from in-person service delivery.**Health service delivery outcomes:** Telemedicine services for children and adolescents with chronic illnesses are feasible and cost-effective.
Van den Berg et al.^44^	2012	**Patient outcomes:** Telemedicine (including video) aids self-management and leads to better behavioural changes (e.g. diet, exercise, self-efficacy) and quality of life. However, some studies (26/68) excluded patients with cognitive, visual, and auditory impairments, making it difficult to generalise the findings of the review to these sub-populations.**Health service delivery outcomes:** This modality appears economically beneficial in terms of reducing healthcare costs and hospitalisation rates.
Wile and Pringsheim^24^	2013	**Patient outcomes:** Telemedicine (including video) and in-person care improved tic severity compared with baseline measures for those living with Tourette’s Syndrome. No conclusions could be made about the efficacy of each mode of treatment delivery or equivalence ‘due to lack of inactive control’ (p. 391).
Zhai et al.^27^	2014	**Patient outcomes:** The authors observed a nominal but statistically significant effect on decreased levels of glycated haemoglobin for patients with type II diabetes mellitus. While telemedicine (including video) appears promising for the management of this condition, the authors detected a high degree of publication bias.**Health service delivery outcomes:** Due to small samples and heterogeneous data, no conclusions about cost-effectiveness could be drawn.

## Patient outcomes

A review of telecounselling for depression pooled results from 498 adults of African-American, Spanish, and Asian origin and found some evidence of increased satisfaction among individuals from ethnic minority communities. Limited data also pointed towards longer-term health benefits for these patients.^30^ The review of telepsychiatry analysed results from a total of 1054 patients from psychiatric services and concluded that telepsychiatry is safe to use. However, there was insufficient evidence regarding its effectiveness in the routine management of mental health patients.^31^ Furthermore, a review of videoconsulting for depression found it to be as beneficial as in-person care.^32^

Overall, two reviews indicated that a good therapeutic alliance between clinician and patient is possible via video,^33,34^ but no improvement in health outcome was found in one of these reviews.^33^ There were two reviews that indicated that this modality may be better than in-person care for some conditions, such as autism^26^ and anxiety-related disorders.^19^ This was often attributed to the difficulties and low motivation that can sometimes accompany these conditions, and may hinder engagement with alternative forms of intervention. The feasibility, acceptability, and sustainability of telemental health for children and adolescents have also been reported. Overall, two reviews found that telemental health assessment with this group of patients was, in general, reliable and feasible.^20,23^

In the review of the use of telemedicine in diabetes, 23 of the 27 randomised controlled trials reported improved metabolic outcomes. In total, 12 of the 23 studies produced significant results, while only two observed negative health outcomes.^35^ Another review of 19 studies using videoconferencing in oncology found no conclusive evidence of a difference between video consultation and face-to-face consultation.^36^ However, a review of telemedicine for asthma concluded that there was there was a reduction in hospital admissions.^37^ Some reviews found differences in outcome depending on the communication medium. For example, a review of telemedicine for heart failure concluded that videophone did not improve outcomes but structured telephone follow up and telemonitoring did, including all-cause mortality.^28^ In another review of the use of digital communication between clinicians and young people requiring mental healthcare, 5 of the 12 studies concerned videoconferencing, but significant improvement in health outcome was only seen with email contact. However, the authors concluded that the evidence dealing with email and web-based discussion was more reliable and rigorous than for videoconferencing.^38^

Only one review of the clinical use of Skype was identified.^7^ Arnfield and colleagues summarised evidence from the 27 published studies and concluded that 26 of the 27 articles presented results that were supportive of Skype. In particular, Skype was adequate for patients across the age spectrum, although the majority of studies described applications involving adult patients. Overall, five studies concluded that Skype offered good communication between patients and clinicians. However, concerns about the security and privacy were raised in the majority of the included papers.

## Healthcare professional outcomes

Overall, four reviews considered the health professional perspective in more depth. A review of forensic telepsychiatry in mental health reported that healthcare professionals were less satisfied with using videoconferencing for the purpose of assessment compared with the prisoners they were treating.^39^ This is acknowledged in another review that found professionals preferred using email and other web-based approaches to videoconferencing when communicating with young people with mental health disorders. Emails were viewed as beneficial to therapy because written communication allowed clinicians to recount the young person’s personal and health experiences.^40^ Schlegi and colleagues found that videoconferencing provides little benefit for clinical staff in terms of cost or time savings, but may assist patients who live in remote places to access specialist psychological service.^41^ Weger and colleagues concluded that the studies tend to highlight that health professionals are more reluctant than the service users to use the technology.^19^

## Health service outcomes

Health service delivery outcomes represented an outcome of interest in 30 reviews. In the review on the use of telemental health, Hilty et al. reported reduced length of hospitalisation and better medication adherence.^26^ Although the sub-analysis of a larger systematic review^28^ and meta-analysis into heart disease^42,43^ found that structured telephone follow up and telemonitoring reduced heart failure-related hospitalisation admittances, the authors found no conclusive evidence that this occurred with videoconferencing. In another review on the use of telemedicine for older patients, 36 of the 50 studies (that included a medical endpoint) comprised various health service outcomes (i.e. cost, hospitalisation, healthcare utilisation).^44^ The conclusion of the review was that video consultation may be an effective method for decreasing healthcare expenditure. A review focused on telepsychiatry reported that there were limited data in support of the cost-effectiveness of video technologies.^45^ Another review of internet videoconferencing for long-term conditions reported similar findings about the evidence for cost-effectiveness.^46^ Armfield and colleagues reported that Skype was more economical than face-to-face appointments with savings accruing from avoided travel,^7^ while the review of Peeter et al. of the financial benefits of videoconferencing in comparison with usual care at home reported no advantages compared with usual care.^47^ Taking into consideration the limited data surrounding the financial implications of telemedicine, some reviews were unable to form any meaningful conclusions about its cost-effectiveness.^27,40,48^

## Summary of the findings

In the home setting, for patients with long-term conditions, the review of reviews indicates that there is no formal evidence in favour of or against the use of internet videoconferencing. Evidence for its impact on health outcomes suggests it mostly has equivalence with face-to-face communication. The evidence for equivalence seems to be the strongest in mental health conditions. Furthermore, internet videoconferencing seems to be an acceptable mode of care delivery for patients with long-term conditions. Research indicates that patients who have experienced videoconferencing with clinicians, like it. However, there is limited evidence about healthcare professionals’ satisfaction with this mode of communication. Little is also known about the impact of videoconferencing on health service costs. The discussion sections of most reviews often suggest that further research is needed around cost, ethics and safety, and the practical challenges when implementing internet videoconferencing. Finally, this review of reviews identified only one review of the clinical use of Skype. Many of the reviews identified included internet videoconferencing as one of a number of communication channels with the patient, making it difficult to disentangle the actual impact of videoconferencing.

In the remainder of this paper we explore where, in the patient pathway, videoconferencing could be used to deliver healthcare and offer advantages to patients, their clinicians and the healthcare system.

## NICE pathways review

### Methods

We undertook a review of NICE pathways for the diagnosis, treatment and management of long-term conditions for children, young people and adults. We chose to look at psychosis and schizophrenia, HIV, diabetes, liver fibrosis, eczema, psoriasis, cystic fibrosis, cancer, asthma, arthritis, kidney and sickle cell disease specifically as these were the conditions we researched in the LYNC study project.

Using the online NICE guideline pathway tool (available at www.nice.org.uk/guidance/published?type=apg,csg,cg,mpg,ph,sg,sc), we searched the guidance and advice list for the chosen conditions. For each condition, two reviewers (clinician and researcher) read all the statements and related quality standards, and noted whether videoconferencing could be used as a mode of healthcare delivery during the implementation of the guidelines. This involved going through the NICE interactive flowchart and examining all the recommendations. Depending on the condition, the flowchart covered information on preventing, detecting, diagnosing, monitoring and managing the long-term conditions in primary, secondary and community care. It also included principles of care and general statements about the quality of support provided to patients. We then compared our findings to those of the review of reviews and the LYNC study to further illustrate the practical applications of videoconferencing in clinical practice.

## Results

Our assessment of NICE pathways suggests that Skype and other forms of internet videoconferencing could be used to review, monitor and plan care for patients with long-term conditions (Table 4). In line with the current evidence and guidelines, internet videoconferencing could be implemented to help clinicians support patients through:

**Table 4:** Findings from the review of NICE guidance for chosen long-term conditions.

Long-term condition	Summary of NICE guidance for clinicians	Opportunities to include videoconferencing as part of care	Benefits and challenges	Evidence from the review of reviews	Lessons from the LYNC study
Psychosis and schizophrenia	**Assessment in specialist mental health services:**If a clear diagnosis of psychosis cannot be made, monitor regularly for further changes in symptoms and functioning.**Care planning in specialist mental health services:** Agree a suitable time to review the care plan.Support patients to develop strategies, including risk- and self-management plans, to promote and maintain independence and self-efficacy, wherever possible.**Communication and information:**When communicating with patients, use diverse media, including letters, phone calls, emails or text messages, according to their preference.	Monitoring, reviewing and support could be provided using Skype and other forms of videoconferencing rather than face-to-face.	Benefits for patients: reduced need to travel; saved time; improved patient engagement and access to healthcare services.Challenges: asynchronous modes of digital clinical communication, such as text messaging, may be more appropriate; ethical issues around privacy (in terms of information shared and surroundings) and informed consent; issues around reliability of the internet connection, payment for services, cost to the patient and challenges surrounding information governance.	Patients are satisfied with using videoconferencing in order to discuss their mental health conditions with a professional.^18,31,45^ Videoconferencing can be successfully used to provide educational services to patients.^31^No loss of therapeutic alliance.^20,22^Evidence suggests that the distance between patients and healthcare professionals could reduce anxiety and over-simulation.^21^	Asynchronous modes of digital clinical communication, such as text messaging, may be more appropriate for providing support; privacy and safety issues; possible increased dependence on the clinician due to increased and easy access to information.
Diabetes	**Individualised care:**Adopt an individualised approach to diabetes care that is tailored to the needs and circumstances of patients with type 2 diabetes, taking into account their personal preferences, comorbidities, risks from polypharmacy, and their ability to benefit from long-term interventions.**Patient education and lifestyle advice:**Offer patients with diabetes and their family members or carers (as appropriate) a continuing programme of education for diagnosis.Tailor the education programme to each individual patient and their family members or carers (as appropriate).Provide an alternative to the education programme of equal standard for a person unable or unwilling to participate in group education.	The programme of education could be delivered via Skype and other forms of videoconferencing rather than face-to-face.	Benefits to patients: reduced need to travel; saved time; group and individual educational programmes possible: overcoming illness issues; improved patient engagement and access to healthcare services.Challenges: training in technical use of equipment may be best delivered face-to-face; ethical issues around privacy (in terms of information shared and surroundings) and informed consent; issues around reliability of the internet connection, payment for services, cost to the patient and challenges surrounding information governance.	Videoconferencing is a promising tool in the management of patients’ glycaemic levels.^73^Patient satisfaction with videoconferencing is high.^35^This modality appears to reduce travel and in-clinic waiting time.^35^	Digital clinical communication can be used to support patients in maintaining good control of diabetes and prevent or delay long-term complications. Asynchronous modes of digital clinical communication, such as text messaging, may be more appropriate for providing quick advice on changing dosages; possible increased dependence on the clinician due to increased and easy access to information.
Dermatology: psoriasis, eczema	**Principles of care:**Offer people with any type of psoriasis support and information tailored to suit their individual needs and circumstances, in a range of different formats, so they can confidently understand their diagnosis and treatment options, how to use prescribed treatments safely and effectively; when and how to seek further general or specialist review; strategies to deal with the impact on their physical, psychological and social wellbeing.**Assessments:**Offer annual assessment for psoriatic arthritis to people with any type of psoriasis.Discuss risk factors for cardiovascular comorbidities with people who have any type of psoriasis. Where appropriate, offer preventative advice, healthy lifestyle information and support for behavioural change tailored to meet the needs of the individual.**Topical therapy for psoriasis:**Offer practical support and advice about the use and application of topical treatments.	Assessment, support, advice and information could be provided via Skype rather than face-to-face.	Benefits to patients:reduced need to travel; saved time; possibility of regular reviews of the condition; improved patient engagement and access to healthcare services.Benefits to clinicians: saved time.Challenges:Safety issues around diagnosis over video; ethical issues around privacy (in terms of information shared and surroundings) and informed consent; issues around reliability of the internet connection, payment for services, cost to the patient and challenges surrounding information governance.	No specific reviews published on videoconferencing and dermatology since 2009.	Digital clinical communication can be used to monitor the progression of the condition and alleviate psychological distress; possible increased dependence on the clinician due to increased and easy access to information.
Cancer	**Quality statements:** Patients with cancer (and their families and carers) should have their psychological and social needs assessed at key points on their care pathway and receive support based on their identified needs.Treatment, care and support, and the information given about cancer, should be both age-appropriate and culturally appropriate. It should also be accessible to people with additional needs such as physical, sensory or learning disabilities, and to people who do not speak or read English.	Support and information could be provided via Skype rather than face-to face.	Benefits to patients: reduced need to travel; saved time, particularly when the condition is rare and the clinic far away from the patient’s home; interpreter including signer for hearing disabled could be involved remotely; group and individual support possible; improved patient engagement and access to healthcare services.Challenges: ethical issues around privacy (in terms of information shared and surroundings) and informed consent; issues around reliability of the internet connection, payment for services, cost to the patient and challenges surrounding information governance.	Evidence suggests videoconferencing is feasible and effective for assessing, monitoring, and managing patients with cancer.^36^Patient satisfaction is high.^36^	Digital clinical communication can be used to enable patient access to expert care and support alongside up-to-date treatment. Asynchronous modes of digital clinical communication may be more appropriate, particularly if the patient values privacy and ability to contact the healthcare professionals without having to wait until the next appointment; possible increased dependence on the clinician due to increased and easy access to information.
Cystic fibrosis	**Information and support:**Provide people who are newly diagnosed with cystic fibrosis and their family members or carers (as appropriate) with opportunities to discuss their concerns.**Managing cystic fibrosis:**Offer people with cystic fibrosis a comprehensive annual review.**Preventing cross-infection:**Inform people with cystic fibrosis, their family members or carers (as appropriate) and staff involved in their care about the risk of cross-infection and how to avoid it.	Support and information could be provided via Skype rather than face-to face.Reviews could be undertaken via Skype to avoid cross-infection.	Benefits to patients: reduced need to travel; saved time; possibility of regular reviews of the condition; improved patient engagement and access to healthcare services.Challenges: ethical issues around privacy (in terms of information shared and surroundings) and informed consent; issues around reliability of the internet connection, payment for services, cost to the patient and challenges surrounding information governance.	No specific reviews published on videoconferencing and cystic fibrosis since 2009.	Digital clinical communication can be used to maintain health status, prevent decline, and for peer support. Asynchronous modes of digital clinical communication may be better used for urgent matters; possible increased dependence on the clinician due to increased and easy access to information.
Arthritis	**Education and self-management:**People with rheumatoid arthritis are offered educational and self-management activities within 1 month of diagnosis. It is essential that the offer of educational and self-management activities is not a 'one-off', but is repeated throughout the course of the disease to ensure patients have the opportunity to participate at a time, individual to them, that will support them to derive the greatest benefit.**Monitoring:**Offer people with satisfactorily controlled established arthritis review appointments at a frequency and location suitable to their needs.	Education could be offered via Skype rather than face-to-face.Repeated appointments could be offered via Skype rather than face-to-face.	Benefits to patients:reduced amount of travel, particularly if mobility is an issue; saved time; improved patient engagement and access to healthcare services.Challenges: ethical issues around privacy (in terms of information shared and surroundings) and informed consent; issues around reliability of the internet connection, payment for services, cost to the patient and challenges surrounding information governance.	No specific reviews published on videoconferencing and arthritis since 2009.	This clinic did not use digital clinical communication at the time of the study; possible increased dependence on the clinician due to increased and easy access to information.

NICE: National Institute for Health and Clinical Excellence.

1. Advice and education: supporting patients in developing strategies to promote and maintain independence and self-efficacy.
2. Information: signposting and providing patients with information about their condition.
3. Relationships: enabling and helping to maintain better communication, and supporting patient and their family’s psychological and social needs.

There is a range of clinical, supportive, educational and administrative functions for which internet videoconferencing may be useful, from supporting care planning and monitoring, organising follow-up consultations, reviewing or adjusting medication to providing group or individual educational programmes for patients with diabetes. The findings from our assessment suggest that videoconferencing would be a particularly useful mode of communication when considering the NICE general principles of care and quality statements underpinning clinical practice, most notably, those around offering support and information on individual patient needs and circumstances.

Videoconferencing could be implemented to meet patient need and preference at the appropriate time and for specific reasons. Given that the main advantage of videoconferencing is its convenience, the wider literature indicates that the implementation of this method could potentially reduce barriers to treatment. For example, patients may be able to save time travelling to the clinic and, in doing so, avoid disruption to their daily activities. Patient education, either group or individual, could be undertaken using internet videoconferencing. Studies that have used videoconferencing for patient education report a number of advantages, including patient satisfaction, improved health outcomes and overcoming mobility and illness issues.^49–51^ Care planning, reviews and monitoring, especially for those patients that have considerable distances to travel for their appointments or are not well enough, could be assisted using videoconferencing. Examples from our own LYNC research suggest that one of the key benefits of digital clinical communication in comparison with face-to-face care was that it improved access to health services and increased patient engagement. Young people highlighted the role that digital clinical communication played in saving them time, they did not have to fit around the operating times of the clinic and were able to have a more frequent contact with their healthcare professionals. Digital clinical communication was thought to help reduce the power imbalance in the patient–clinician relationship, with clinicians fitting into the young person’s world rather than the young person being expected to fit into the clinical world. As a result, young people believed they had received a more personalised care tailored to their own preferences and healthcare needs. Both patients and clinical team members noted that this improved the relationship between patient and clinician, and prompted better control of young peoples’ condition levels of self-care. A number of issues contributed to this perceived improved personal relationship and self-management: the ability to have more frequent contact with a specific clinician who is known to the patient and likely to know that particular young patient’s personal circumstances and what is important to them; the ability to have questions or queries answered quickly and therefore take better control of the condition; and the ability to communicate with clinical teams between appointments.

Overall, the review of NICE pathways points to a number of opportunities for adoption or expansion of internet videoconferencing for patients with long-term conditions. However, the opportunities to use this mode of communication and the actual benefits derived from it are likely to depend on many factors. The focus on the patient experience is important but this experience may vary substantially according to the treatment pathway. Choosing to utilise internet videoconferencing may be influenced by patients’ preference, their digital resources and skills, clinician’s motivation to use it, the organisational and resource considerations, the healthcare setting and the actual long-term condition. The LYNC study found that these factors were likely to impact on the implementation of digital clinical communication, but could be minimised if the clinician is clear on what they hope to achieve in terms of the ability of the patient to manage their condition and health outcome. Furthermore, adoption of internet videoconferencing is likely to vary based on factors such as patient group or clinical setting. For example, communication problems in the elderly patients, such as visual or hearing impairment, are likely to impact the use of videoconferencing. In our study, the use of different modes of communication varied across clinical teams and according to the reason for making contact. Digital modes of communication also appeared to work best for patients and clinicians who had pre-existing and established relationships.

Despite these insights, the use of videoconferencing in line with the current guidelines for patient care may entail a number of challenges. Issues such as reliability of the internet connection, privacy in terms of surrounding environment, confidentiality of the information shared and informed consent, support for patients and clinicians using the technology, cost to the patient, and challenges surrounding information governance, compliance with legal and regulatory standards around privacy and data protection are often mentioned in the literature, but rarely explored in detail. The lack of data and the need to consider not only costs to health services, but also costs to patients and their social networks, are often highlighted as an important issue to explore.^52^ Furthermore, the use of videoconferencing raises a number of ethical questions. In the LYNC study, young people had different understandings of confidentiality and privacy than clinicians, and expressed different levels of concern about possible breaches.^53^ Clinicians emphasised the importance of informing patients clearly about the implications of using digital communication and seeking their consent prior to commencing this service. However, they also expected that patients will take responsibility for knowing the risks. There are inherent challenges around the roles and responsibilities of clinicians when using videoconferencing for consultation purposes. With a growing number of opportunities for adoption and expansion of videoconferencing, further research exploring the actual implementation challenges to inform and support the development of services is also needed. If internet videoconferencing is to be used in line with the current guidelines for patient care, the risks and benefits for different patient groups and healthcare settings ought to be explored.^54,55^

## Discussion

This review of reviews identified and synthesised a body of literature relating to the use of internet videoconferencing between patients with long-term conditions and their treating clinicians from the patient’s own home. The review indicated that there was no formal evidence in favour of or against the use internet videoconferencing. A total of 6 out of 35 review articles included in this review concluded that patients were satisfied with the use of videoconferencing. However, there was limited evidence that its use led to a change in health outcomes. In some cases, it compared unfavourably with other methods of communication, such as web or telephone-based communication. Evidence of healthcare professional satisfaction when using this mode of communication with patients was limited. Little was also known about the impact of videoconferencing on health service cost, ethics and patient safety.

To our knowledge, this is the first review of reviews conducted in this topic area. However, in comparing our findings with the systematic reviews of internet videoconferencing in areas other than chronic conditions, it appears that some findings are comparable. For instance, research has shown that such modes of communication are acceptable to patients and effective for support after premature birth,^56^ follow up after total joint arthroplasty^57,58^ and care for paediatric patients with various healthcare conditions.^59^ Good evidence from reviews also exists for other settings, some forms of videoconferencing have been found to be feasible and acceptable for patients based in hospitals, clinics or nursing homes.^60^ However, the reviews also highlight that despite a number of studies on videoconferencing, high-quality evidence is only beginning to emerge. In this review, we identified a considerable number of reviews that were of lower quality. Limited details of included studies’ characteristics, potential biases in the selection of articles and the methodological limitations of primary studies included in the reviews are often cited as difficulties in bringing the conclusions together. Many of the reviews in our review focused on various internet videoconferencing modes and included them as one of a number of communication channels studied, providing little evidence on the actual impact of videoconferencing. Publication bias might also be a potential issue. Videoconferencing technology can often be implemented and used in routine healthcare practice, but the evidence of its acceptability and efficacy may not be published in academic literature. As such, and despite a substantial increase in the number of published papers on videoconferencing in the last few decades, further research on its deployment is needed. Our review of reviews adds to the knowledge by summarising the extent, range, and nature of findings of many separate reviews.

In the UK, there is a strong policy and political push towards the use of digital technologies for clinician–patient communication to improve and transform care models and patient pathways. Software has been rolled out to allow Skype to be used safely and securely in the specialist clinical settings. The evidence base for the clinical use of Skype is growing, our review of reviews concluded that Skype offered good communication between patients and clinicians and was more economical than face-to-face appointments with savings accruing from avoided travel. The review of NICE pathways points to a number of opportunities for adoption and expansion of Skype and other forms of videoconferencing for patients with long-term conditions. However, as the review and the lessons from the LYNC project highlight, implementing internet videoconferencing services poses a number of challenges. Past research suggests, for example, that video consultations can be less reliable^61,62^ and less safe^63^ in comparison with face-to-face meetings. Issues of privacy, confidentiality and informed consent, support for patients and their clinicians, and challenges around information governance and compliance are often explored superficially. Very few studies have looked at how they are interconnected and impact on implementing the videoconferencing services.^54,55^ Internet videoconferencing for patient–clinician consultations also requires some basic necessary technical conditions to be in place, including basic technological infrastructure and available technical support. Recent research shows that the quality of videoconferencing can be affected due to failed calls and audio/video jitter.^64^ The evidence of the practical issues when introducing video consultations from the UK is only beginning to emerge, suggesting that establishing such services is complex because of disruption of routines in traditional clinic, and real and perceived information governance issues (see, for example VOCAL study findings^54^). The introduction and implementation of videoconferencing services inevitably changes the clinical and administrative ways of working. The lack of training and support for clinicians and patients are often cited as barriers to the use of videoconferencing.^65,66^

Furthermore, with non-secure modes of communication such as Skype, the challenges surrounding information governance and compliance with legal and regulatory standards around privacy and data protection have further implications for how the communication is undertaken. There are inherent risks and ethical issues for clinicians and patients, not least around finding appropriate physical space for the video interaction and ensuring that that the technology works. The clinicians need to be aware of the risks associated with use of non-secure modes and make best efforts to minimise these risks, while also ensuring that the patient also understands the risks of sharing information in this way.

Finally, the research on internet videoconferencing is rapidly changing, terminology and modes of communication are becoming established and increasingly used in routine practice. Studies that have examined longitudinal changes in content within the internet videoconferencing literature show that publications on such modes of communication have moved from technical aspects to focusing more on patients and clinical applications.^67^ While current evidence base for the use of videoconferencing is equivocal, it is likely to change as more research is undertaken and evidence published. With more videoconferencing services added in more contexts, research needs to explore how internet videoconferencing can be implemented in ways that it is valued by patients and clinicians, and how it can fit within organisational and technical infrastructure of the healthcare service.

### Limitations of the study

Our review of reviews had some limitations. In order to retrieve a manageable number of records with a high chance of relevance, search terms focused on the specific technology of videoconferencing. More general terms, such as telehealth and remote consultation, that may be used to describe the use of a wide range of remote communication tools in this context, including videoconferencing, telephone, email, etc. (and are therefore also likely to retrieve a large number of irrelevant records), were not included. There is no guarantee that all relevant systematic reviews were retrieved. However, the use of the MeSH term ‘videoconferencing’ in the search, which was first introduced to *MEDLINE* in 2005, will have helped to retrieve some relevant records that would otherwise have been missed. What is more, conducting a review of existing reviews presents challenges in synthesising large amounts of information. In this review, we aimed to summarise the existing literature, while also taking into account the quality of each of the review. We are conscious that summarising reviews can lead to a loss of information. For example, a large number of the reviews in our review focused on various internet videoconferencing modes. We did not differentiate between secure and non-secure platforms when reporting our findings. Therefore, our results are only a high-level summary.

In addition to our review of literature, this study examined the NICE guidelines and identified where, in the patient pathway, the use of videoconferencing might be possible and of advantage. However, the pathway review presented challenges too. While the NICE pathways are generally considered a way of implementing evidence-based guidelines into practice, they are recommendations and should be used in conjunction with practitioner’s judgement and discussion with people using services. Therefore, we recognise that we may not have been able to summarise all the clinical functions for which internet videoconferencing may be useful, at best, the review reflects the possible opportunities for adoption or expansion of this mode of communication for patients with long-term conditions.

## Conclusions

The evidence base for the use of internet videoconferencing for consultation between a healthcare professional and patient is growing, but there is still a lack of clear evidence relating to the impact on outcomes and cost. While internet videoconferencing appears to be feasible and acceptable to patients, there are unanswered questions about the ethics of these consultations and the actual implementation challenges.

## Supplementary Material

Supplementary material
