# Supplementary material for: Internet videoconferencing for patient–clinician consultations in long-term conditions: A review of reviews and applications in line with guidelines and recommendations
Source: Digit Health. 2019 Apr 23;5:2055207619845831. doi: 10.1177/2055207619845831 (PMC6495459; doi:10.1177/2055207619845831)
Supplement: Supplementary material [file 845831_Suppl_file_1.pdf]

Supplementary file 1: Search strategies

Searches for Skype review of reviews

Search within EndNote results for LYNC project review of systematic reviews (search date 09/05/14)

	Any field	Contains	skype	
Or	Any field	Contains	videoconferenc*	
Or	Any field	Contains	Video-conferenc*	
Or	Any field	Contains	Google AND (talk or hangouts)	

Total: 91
Total 2009 onwards: 78

2017 update of skype element of search for review of systematic reviews
Ovid MEDLINE(R) 1946 to May Week 4 2017, searched 07/06/2017

1	(video-conferenc* or videoconferenc* or videophone* or video-phone* or video chat or video call* or Voice over Internet Protocol or VoIP or skype or (google adj2 (talk or hangouts)) or facetime or zoom or gruveo or gotomeeting or amazon chime or cisco webex or teamviewer or apache openmeeting or oovoo or whatsapp or talky or viber or facebook messenger or tango or wechat or kakaotalk or justalk or nhs one or nhsone or appear?in or join?me or voca).tw.	3870
2	Videoconferencing/	1184
3	1 or 2	4278
4	(metaanalys* or "meta analys*" or "meta-analys*").tw.	92450
5	"systematic* review*".mp.	83679
6	meta analysis.pt.	80909
7	4 or 5 or 6	160929
8	3 and 7	64
9	limit 8 to ed=20140510-20170607	22

2017 search for skype terms limited to publication type review
Ovid MEDLINE(R) 1946 to May Week 4, searched 07/06/2017

1	(video-conferenc* or videoconferenc* or videophone* or video-phone* or video chat or video call* or Voice over Internet Protocol or VoIP or skype or (google adj2 (talk or hangouts)) or facetime or zoom or gruveo or gotomeeting or amazon chime or cisco webex or teamviewer or	3870

	apache openmeeting or oovoo or whatsapp or talky or viber or facebook messenger or tango or wechat or kakaotalk or justalk or nhs one or nhsone or appear?in or join?me or voca).tw.	
2	Videoconferencing/	1184
3	1 or 2	4278
4	limit 3 to "review articles"	397
5	limit 4 to yr="2009 -Current"	212
